# Cognitive and emotional reactions to pictorial-based risk communication on subclinical atherosclerosis: a qualitative study within the VIPVIZA trial

**DOI:** 10.1080/02813432.2023.2178850

**Published:** 2023-02-28

**Authors:** Elin M. Andersson, Helene Johansson, Steven Nordin, Kristina Lindvall

**Affiliations:** a Department of Psychology, Umeå University, Umeå, Sweden; b Department of Epidemiology and Global Health, Umeå University, Umeå, Sweden

**Keywords:** Cardiovascular disease, decision making, health behaviour, prevention, qualitative content analysis

## Abstract

**Objectives, setting and subjects:**

Atherosclerosis screening with ultrasound is non-invasive and can be used as part of risk communication. The potential of personalised and pictorial-based risk communication is assessed in VIPVIZA, a population-based randomised controlled trial that aims at optimising cardiovascular disease (CVD) prevention by investigating the impact of visualisation of subclinical atherosclerosis. The present aim was to explore cognitive and emotional reactions evoked by the intervention as well as attitudes to any implemented life style changes in VIPVIZA participants in the intervention group with improved health status and furthermore to study possible interactions between these factors. Understanding mechanisms of action was central since non-adherence to preventive guidelines are often faced in clinical practice.

**Design:**

In-depth interviews with 14 individuals were analysed with qualitative content analysis.

**Results:**

Cognitive and emotional processes were highly interlinked and described by the main theme Cognitive and emotional reactions in strong interplay for orchestration of health oriented behavioural change. The informants’ descriptions revealed two distinctly different psychological processes which constituted the two subthemes, Problem-focused coping and Encouragement-driven process.

**Conclusions:**

The results highlight that an interaction between emotional reactions and efficacy beliefs is important in facilitating behavioural change. Furthermore, the results underscore the importance of the risk message being perceived as clear, accurate, reliable and also emotionally engaging and thereby show why atherosclerosis screening and pictorial-based risk communication have the potential to contribute to effective CVD prevention strategies and shared decision making in primary care. **Trial registration:** ClinicalTrials.gov identifier: NCT01849575, registration 8 May 2013.

**Key points**
- Atherosclerosis screening and pictorial-based risk communication have the potential to contribute to more effective CVD prevention strategies.
- Risk messages on atherosclerosis status were perceived as clear, accurate, reliable and emotionally engaging.
- An interplay between efficacy beliefs and emotional reactions facilitated behavioural change.
- Patients’ understanding of CVD risk is important for shared decision-making and of relevance for non-adherence to preventive guidelines.

## Introduction

The world is facing major challenges regarding health prevention of life style related chronic diseases and substantial medical costs. Non-adherence to preventive guidelines is of great concern [1]. In the literature evaluating the impact of global CVD risk assessment in primary prevention, one important focus, apart from clinical outcomes, is the accuracy of perceived risk [2]. That is, whether an intervention using a risk score instrument improves participants’ ability to report an estimate of their personal risk. However, improved accuracy of perceived risk is not equivalent to whether a risk is considered to be relevant, emotionally engaging or useful for health-oriented decision making. Support for personalised risk communication as a way to motivate lifestyle changes was found to be weak in a systematic review of systematic reviews, whereas use of pictorial techniques such as ultrasound was found to be the most promising method of communicating risk [3]. Ultrasound techniques enable investigation of CVD risk by assessment of atherosclerosis while still asymptomatic, but support for its role in CVD prevention is inconsistent [4,5]. Early stages of atherosclerosis are asymptomatic and are typically not diagnosed. Fortunately, severe atherosclerosis can be slowed down and even reversed [6]. Screening for subclinical atherosclerosis is motivated since score instruments may underestimate the risk among asymptomatic individuals [7], and since prevention guidelines focus on individuals at high or very high risk of CVD [8], while most cases of CVD occur outside the high-risk group. Atherosclerosis screening with ultrasound is non-invasive, and can be accessible also at long distances from hospitals (small portable machines), which enables population screening. Furthermore, atherosclerosis screening can be used as part of risk communication [9,10].

The Visualisation of Asymptomatic Atherosclerotic Disease for Optimum Cardiovascular Prevention (VIPVIZA) trial assesses the impact of visualisation of asymptomatic atherosclerosis, added to traditional statistical risk factor-based risk communication. Since the VIPVIZA study provides evidence of the contributory role of pictorial presentation of atherosclerosis for reduction of CVD risk factors [9,11], it is highly relevant to assess how participants perceive, understand and make sense of the risk communication to gain understanding of components of the intervention and mechanisms underlying lifestyle modification.

To the best of our knowledge, there is no qualitative study that has explored cognitive and emotional reactions to personalised and pictorial-based CVD risk communication, focusing both on present and previous experiences.

Applying a qualitative approach, our study assessed VIPVIZA participants in the intervention group who at baseline and three-year follow-up had received a pictorial ultrasound-based risk message about presence of atherosclerosis. The aim was to explore cognitive and emotional reactions evoked by the intervention as well as attitudes to any implemented life style change in VIPVIZA participants in the intervention group with improved health status, and furthermore to study possible interactions between these factors. More specifically, the research questions regarded how the informants described their (a) cognitive and emotional reactions to the pictorial-based intervention, (b) perspectives, such as response efficacy, regarding any implemented lifestyle change and (c) cognitive and emotional reactions in the process of changing or maintaining health behaviour.

## Method

### Study context, the VIPVIZA trial and design of the present study

The VIPVIZA trial, a pragmatic, open-label, randomised controlled trial with masked evaluators (PROBE) is conducted in Västerbotten county in northern Sweden. VIPVIZA is integrated in the Västerbotten Intervention Program (VIP), which offers individual health promotion counselling to all inhabitants of Västerbotten the year they turn 40, 50 and 60 years (*n* = 6500–7000 per year) [12]. Participation rate for VIP during the inclusion period was 68%, and only small social selection bias has been observed [13]. Around 60% of VIP participants have at least one conventional risk factor for CVD (e.g. hypertension) and are thereby eligible for inclusion in VIPVIZA. For those aged 60 years, age alone constitutes an inclusion criterion.

#### VIPVIZA

In total, 4177 VIP participants were invited to the VIPVIZA study, and participation rate was 84.6% (*n* = 3532). Recruitment was done during April 2013–May 2016 in different parts of the county to obtain a representative sample. At baseline, and again after three years, presence of carotid atherosclerotic plaque and IMT value were assessed with ultrasound. Sonographers did not provide any feedback to participants.

At baseline and three-year follow-up in VIPVIZA, participants responded to a questionnaire covering life style habits, medication, psychosocial situation and family history of CVD and diabetes. Blood pressure and anthropometric measurements were taken, and blood samples were collected to measure lipids and blood sugar. Participants also responded to validated psychometric questionnaires. The intervention group, and their primary care physicians, received a letter with a pictorial presentation of the ultrasound result.

**Figure 1.**
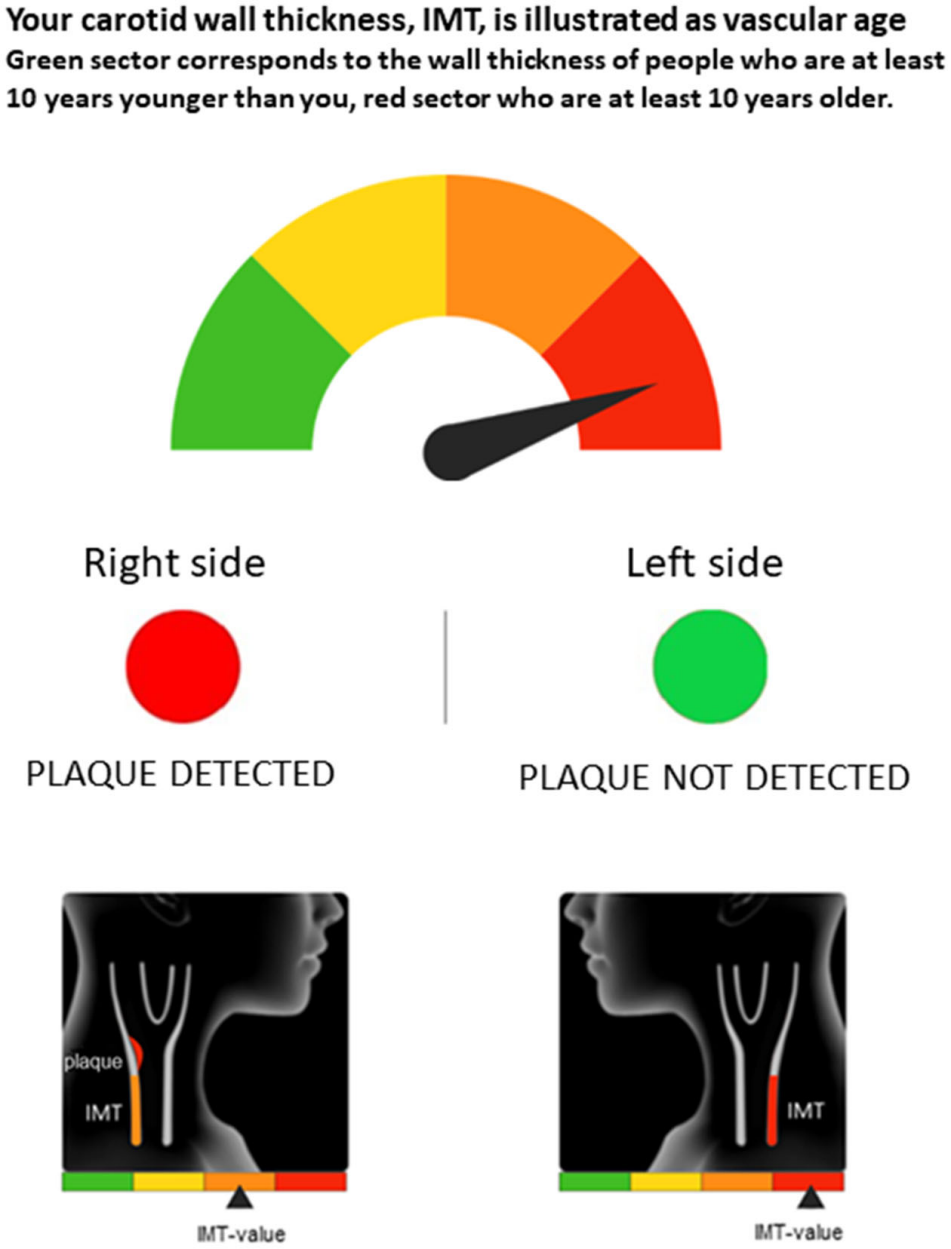
Graphical elements of the result letter, sent to participants in the intervention group after the ultrasound examination of carotid arteries, including information on plaque and intima media thickness.

Intima-media thickness (IMT) was communicated as vascular age, where the individual’s IMT was compared to individuals with the same sex and age in a reference population [9] and depicted as a graphical continuous gauge ranging from green via yellow and orange to red. Green corresponds to the IMT of a person being at least 10 years younger, and red corresponds to an IMT of a person at least 10 years older than the participant’s actual age. Plaque was presented as a traffic light with a red (plaque identified) or green (no plaque) dot. An illustration of graphical elements in the letter is provided in Figure 1. Prior to the study, relevant patient groups were involved in development of the graphical material, and a pilot study was also performed with 95 participants. The information was then adjusted based on participants’ experiences and suggestions to optimise understanding of atherosclerosis as a process and for accurate perception of the ultrasound results. Written information was also provided, describing atherosclerosis as a dynamic process that can be slowed or even reversed by healthier life style and preventive medications (Supplementary Appendix 1). Within two to four weeks after they had received the letter, participants in the intervention group were contacted by a research nurse by telephone for clarifications if needed, any remaining questions and a motivational conversation. In order to evaluate the effect of pictorial-based risk communication, the control group and their primary care physicians did not receive the result from the baseline ultrasound.

Clinical risk factors were measured again at one-year follow-up, and all participants and their primary care physician were given the results. At three-year follow-up, when the participants again were examined with ultrasound and the full baseline routine of validated questionnaires and measurements, the intervention group also responded to questions on reactions to the intervention at baseline. After this visit, all participants received a letter with a pictorial presentation of their ultrasound result. The complete study protocol is available at https://clinicaltrials.gov/ct2/show/NCT01849575.

#### Sampling of informants

All informants were purposively sampled on the basis of their participation in the intervention group and their improved health status. The inclusion criteria were: (i) having received the ultrasound result letter and a nurse call at the baseline visit *or* only having received the ultrasound result letter at the baseline visit, (ii) having received the ultrasound result letter at the three-year follow up, (iii) an absolute reduction of at least 5% in Framingham Risk Score from baseline to one-year follow-up (iv) and unchanged or improved ultrasound result (plaque and IMT-value) at the three-year follow-up. The inclusion did not consider the absolute values at baseline for the Framingham Risk Score, plaque or IMT, but rather *improvement* between baseline and three-year follow-up. The aim of the purposive sampling was also to include informants with a wide range of experiences, and therefore recruit men and women living in urban and rural areas with different educational background and with different health status.

Initially, the belief was that the *combination* of the ultrasound result letter and the follow-up call was important. However, during interviews, it turned out that some informants had not received the follow-up call, and yet had reacted and acted only on the ultrasound result letter. The inclusion criteria were therefore adapted to explore informants’ reactions no matter whether they had reacted to the result letter, the follow-up call or both.

### Data collection and analysis

This study used a combination of an inductive and deductive approach. It was deductive in that the quantitative research on reactions to the VIPVIZA intervention constituted a starting point for the interview guide. During data collection, an inductive approach was instead employed by carefully staying open for the informants’ stories. During analysis, the two approaches were combined into an abductive approach, by altering between ‘theory test’ and explorative assessment of findings.

#### Interview guide

The interview guide was based on relevant constructs identified in the literature (risk perception, self-efficacy, response efficacy, etc). Also, aforementioned quantitative research within VIPVIZA had shown the importance of emotional response. The interview guide is provided in Supplementary Appendix 2. Minor adjustments were made after the first couple of interviews.

#### Procedure

The competing demands of being flexible, yet systematic enough to ask similar questions, were met with a standardised interview guide with open-ended questions and semi-structured interviews. Research nurses selected participants who met the inclusion criteria. After having received their second ultrasound result letter by post, participants were invited by E.A. to a telephone interview. Informants were recruited continuously between October 2018 and March 2019, as closely as possible in timeframe after they had received their letter, and (applicable for individuals with plaque) before any follow-up call with a nurse took place. Two persons declined to participate, who did not seem to deviate from the included informants regarding health status.

E.A. conducted 19 interviews. The first round included 17 interviews, each lasting 30–90 min, all were digitally recorded and transcribed verbatim.

Three informants were excluded after the first round; one informant due to not meeting the inclusion criteria, two due to another diagnosis being more relevant to them to discuss. One informant had strongly contrasting views and reactions (e.g. that the ultrasound result did not reflect personal risk), and was therefore used in the analysis as a *negative case*. A negative case analysis implies assessment of elements of data that do not support or even contradict patterns, and may therefore revise, broaden or confirm the patterns emerging from the data analysis [14]. Since informants were excluded in the first round of interviews, interviews with two additional informants were conducted, but these did not generate substantial new information and were therefore not transcribed. However, these last interviews strengthened the belief that theoretical saturation was reached.

### Ethical considerations

All study participants provided written informed consent when included in the VIPVIZA study and recorded verbal informed consent before their interview. The present study was approved by the Umeå Regional Ethics Board (2011-455-31 M and 2016-245-32 M).

### Data analysis

Qualitative content analysis was applied. In the initial phase the text is shortened into condensed meaning units, which allows for careful assessment of manifest as well as latent content [15]. First, the transcripts were read several times. Then followed a process of identifying *meaning units* on the bases of *content areas*, which, in turn, were identified by combining an inductive and deductive approach. The meaning units were thereafter condensed, coded and grouped into subcategories and categories. An illustration of the analysis process is provided in Table 1. The Open Code software [16] and a specially designed software were used for the analysis. Notes were taken to document questions and insights from the interviews, the transcription phase and during coding. When categories were starting to take form, a negotiation of their content took place between E.A. and K.L. When the level of analysis had reached subthemes and main theme, the results were presented for the interdisciplinary research group with the goal of broadening the preunderstanding by incorporating questions or reflections, and to increase trustworthiness. After this, further negotiation of categories and themes took place between E.A., K.L. and H.L.

**Table 1.** Illustration of the analysing process moving from text to sub-theme.

Text	Condensed meaning unit	Code	Sub-category	Category	Sub-theme
*Well, the thing is that it becomes a warning bell. Yes, well, you have to grab hold of things and not just throw away that piece of paper and think ‘Whatever!’ but instead try to improve things. Yes, I think it has been good.*	Good to get a warning bell and try to grab hold of things and improve them	A strong warning bell	Shocking news	A wake-up call	Problem-focused coping
*I was very tense and hoped, I really hoped for an improved result. But I was worried though that it would not be that way, so the relief was just enormous when I found out that ‘yes, it is in fact improved’.*	The relief was enormous when I saw that it was improved	Relieved to find out	Relived to learn about my result	Joy and relief	Encouragement driven process

*Notes:* The complex nature of the research questions, focusing on cognitive and emotional reactions and their interaction, was one of the reasons why qualitative content analysis was considered an appropriate choice of method. This, in particular, due to how the text in the initial phase is shortened into condensed meaning units, which allows for careful assessment of manifest as well as latent content [1]. In this initial phase, where one should stay close to the original text and shorten the material while still striving to preserve the core meaning, the method was useful for discovering nuances and distinguishing between cognitive and emotional processes. The meaning units were condensed, coded and grouped into subcategories and categories. The analysis continued by going back and forth between codes and subcategories, a process of turning and re-evaluating with the aim of reaching understanding of the participants stories and value codes and categories against the purpose of the study [15].

## Results

Characteristics of informants at baseline are presented in Table 2 for the informants included in the analysis (excluding the negative case, which is described in text separately). Three women and one man were considered to have a high cardiovascular risk at baseline according to Framingham Risk Score, which corresponds to a 20–39% risk of a CVD event or CVD death within 10 years. The other informants had a moderate risk, which corresponds to a 10–19% risk.

**Table 2.** Description of the informants regarding demographic data and atherosclerosis at baseline.

	Men	Women
	(*n* = 7)	(*n* = 6)
Age (years)		
50	2	0
60	5	6
Education		
Basic or middle	5	4
High (university)	2	2
Place of residence		
City	5	2
Rural coastland	0	1
Rural inland	2	3
Plaque status		
No plaque	5	4
Plaque	2	2
IMT colour code		
Green	2	1
Yellow	1	2
Orange	2	1
Red	2	2
Nurse phone call		
Yes	6	4
No	1	2

The analysis of the informants’ cognitive and emotional reactions to the VIPVIZA intervention, as well as attitudes to any implemented lifestyle changes, resulted in one main theme, two subthemes and eight categories. An overview is presented in Table 3. The main theme ‘Cognitive and emotional reactions in strong interplay for orchestration of health oriented behavioural change’ not only incorporates a wide range of different reactions, but also describes how cognitive and emotional reactions are highly interlinked in the psychological processes.

**Table 3.** Overview of results in terms of main theme, sub-themes, categories and sub-categories.

	*Cognitive and emotional reactions in strong interplay for orchestration of health-related behavioural change*		
*Main theme* *Sub-themes*	*Problem-focused coping*		*Encouragement-driven process*
Division of categories in chronological order	Intervention	Reaction	Action
Categories Sub-categories	A source of knowledge Get to know A clear message A source of support The follow-up call: clarifying, reassuring and health promoting Reinforcing messages Recurring feedback Generation of social support	Joy and relief Happy about my result Positively surprised by improved result Relieved to learn about my result A wake-up call Shocking news Unpleasant and disturbing message For me, bad health was remote Empowered and seeing opportunities rather than threats Felt no worries about the result Optimistic about my possibilities for health behaviours I know I can do it (self-efficacy) Habits matter (response efficacy)	Making the decision I realised I have to do something about my health I want to stay in good health Ready for change I have prior experience of disease (own or others) Retirement is a new chapter in life with new opportunities Staying aware, active and self-evaluating After finding out my result, I changed my way of living I have an awareness of health behaviours and CVD Healthy habits are enjoyable and makes me feel well I guess I could have done more for my health Goals and motivators keep me going

First, categories regarding cognitive and emotional reactions and attitudes are described from the perspective of health behavioural change over time, where early stages lay the foundation for later stages. With the first ultrasound result as a starting point, reactions, beliefs and standpoints were evolving over time. Categories are therefore presented in a chronological manner (intervention, reaction and action). Thereafter, the main theme and the two subthemes, which describe two distinctly different psychological processes, are presented.

### Intervention

#### A source of knowledge

Informants had a desire to get to know about their health, and expected, especially when they received their first ultrasound result, to ‘get to know how it really is’. Informants’ understanding of the intervention as a source of knowledge laid the ground for their cognitive and emotional evaluations of their personal health status. The ultrasound result was considered as trustworthy, and several participants used the expression ‘it is stated in black and white…’. The message was clear, definite in nature, convincing, engaging and for real:

Change your habits. You want to see it stated clear and definitive. Everyone knows what to do, really. […] But it is this thing, no two ways about it, I think that’s what I need. To understand that, yes, this is in fact a carrot… [edit: rather than a stick] (Woman, informant 5)

Clarity was central in descriptions of the pictorial message:

Stated clear and definitive, instead of different threshold values and so on. To get a picture is better. (Man, informant no 1)

Furthermore, the nurse was described as a person communicating clear and concrete advice.

#### A source of support

Informants expressed how the intervention had a supportive function for engaging in health behaviours. The role of the nurse is more prominent in this category compared to the previous one: the nurse provided support by explaining the letter, reassured informants if necessary, and gave health promoting advice. A supportive follow-up call could contribute to the informant’s evaluation of response efficacy and self-efficacy:

At first it was like: What? Oh my… but then when it had sunken in, and especially after I had talked to her, I got the feeling that ‘well, this is actually something that you can influence, I can influence this [expressed with emphasis], I'm the only one who can do something about it’ (Woman, informant 13)

To get to know that they were on the right track or that they through their efforts had accomplished something good resulted in encouragement and growing motivation. Social support was also generated, e.g. when life partners got involved in lifestyle changes.

### Reactions

#### Joy and relief

Informants who reacted with joy, relief and/or were pleasantly surprised did not merely do so according to the objective severity of the ultrasound result per se. Expectations did also influence the reactions. Some informants expressed joyful emotions already at baseline because the result was better than they initially had expected:

It said that I was like ten years younger, that my blood vessels looked that good. It was amazing, I couldn’t believe it. (Woman, informant 10)

When informants got their second ultrasound result letter, feelings of joy were often very tangible, in particular among those who had described that they had paid an effort to change their diet and/or exercise routine.

#### A wake-up call

This category expresses reactions of negative valence and includes moderate reactions describing the message as disturbing or unpleasant as well as strong emotions, where the ultrasound result was almost perceived as a shock:

It was exciting and somewhat shocking, after the ultrasound, since it showed that… well, I considered myself to be in good health so it was somewhat shocking to get the letter and finding out that I had plaque, and also that my arteries were thick, they were down for red, so my first reaction was ‘Oh no, I don’t want to die now, I don’t have time for that’. (Woman, informant 13)

As in the case of joyful reactions, negative emotions did not automatically coincide with the objective risk level of the message. Descriptions of the ultrasound result in terms of a wake-up call showed how a reaction to a threatening message was closely related to taking action:

The thing is that it becomes a warning bell. You have to grab hold of things and not just throw away that piece of paper and think ‘Whatever!’ but instead try to improve things. Yes, I think it has been good. (Man, informant 6)

Ill-health was remote for some of the participants, thus they had experienced themselves as being healthy and were therefore negatively surprised:

I found out, stated in black-and-white, that this is how it really is. Me, who consider myself to be very healthy and have more stamina than my colleagues of the same age, and then… then it looks like this. (Woman, informant 13)

Not only plaque but also vascular age significantly affected informants, thus the interpretation ‘older than I actually am’ can sting.

#### Empowered and seeing opportunities rather than threats

Overall, the informants expressed high efficacy beliefs, regardless of the direction of their emotional response. In particular, informants expressed beliefs that health behaviour matters and will be effective, which can be understood as an important prerequisite for behavioural change. The informants also expressed response efficacy beliefs when they learned about their improved results, in relation to implemented lifestyle changes. Self-efficacy was often described in a concrete way, sometimes related to previous experiences, but also prospectively.

Some informants expressed that retirement had or will enable them to be more physically active, eat healthier and perceive less stress. Hindrances, such as cold weather hindering physical activity, were mentioned by a few, but the majority of the informants described that their opportunities for health behaviour were good, and that they saw possibilities rather than threats. Receiving a severe ultrasound result (in this case of plaque on both sides or red IMT) was interpreted as an opportunity to act:

It meant possibilities, definitely. A signal. Because, what now if I had not participated in this? How would it have been in that case? (Woman, informant 6)

### Actions

#### Making the decision

Two distinct views were identified in the informants’ health-related decision-making process. At baseline, roughly half of the informants had a starting point similar to a problem-focused coping process. Central was to try avoiding disease by changing behaviour.

For other informants, the starting point was instead taking actions or deciding to continue with their healthy routine because they wanted to ‘keep a good result’ and stay in good health. The motive was formulated in positive terms; striving for health rather than avoiding disease.

Well, since it looked good, so to say […] I felt that this I have to try to maintain somehow. (Woman, informant 17)

At the three-year follow-up, when the informants found out that their health status had improved, there was a shift among informants who had previously expressed a problem-focused approach to an encouragement-driven approach.

#### Ready for change

Experiences of disease (one’s own or others’) and retirement affected readiness for change. Others’ diseases together with the risk message opened up for thoughts such as ‘it could happen to me’ and motivated lifestyle modification.

I have a childhood friend who died shortly after 60, and then it felt like: No, I want to see my grand children grow up, I want to hang around. […] My boss, she died when she was 61 and I was 60 at that time. So that had just happened [before joining the VIPVIZA study] but, as I said, it becomes a driving force for oneself. (Woman, informant 13)

Experiencing death from CVD in the family could strongly affect readiness for change. After receiving her first ultrasound result, one informant described her view regarding possibilities to affect the risk for CVD as:

My thoughts were that I want to try as much as I can to influence my health so that I don’t get those plaques. I thought that if I can at least stay on this level, then maybe I don’t have to get as sick as for example my father. (Woman, informant 10)

Retirement represented not only new living conditions facilitating health behaviour, but also a new chapter in life, a mental window for change with new opportunities for living a healthy and meaningful life. Holistic views on health were also expressed:

You get the opportunity to steer you own life so to speak, and do whatever you yourself find interesting. And I resumed my old hobby as an amateur musician at that time [when he retired] I think that that in itself also can contribute to improvement, actually. At least an improvement of quality of life, I don’t know if it affects the vessels, but at least it affects onés well-being. (Man, informant 16)

#### Staying aware, self-evaluating and active

This category includes increased awareness of disease and a changed mindset regarding food choice and exercise, setting goals for health, and positive attitudes to health behaviours. Informants described changes in diet and increased physical activity. Several talked about what they had accomplished (e.g. weight loss), and gladly told how it was done. Some had also started taking medication, and expressed that they were motivated to do so and trusted their physician’s advice. Several informants described an increased awareness. For some it was pronounced (e.g. searching for a certain label for healthy food when shopping groceries), whereas others described that healthy living is something they ‘have in mind’.

Overall, healthy habits were enjoyable, and made informants feel well and good about themselves. Some informants were physically active before the intervention, whereas others started after they had been included in the VIPVIZA study. Appreciating healthy habits seems to have facilitated staying aware and active:

We have changed quite a lot of things, [e.g. buying groceries with attained recipes for home delivery] and I must say that is actually great fun. There are many great dishes that I would never have thought that I would like, and I have never really appreciated vegetables but now I have managed to get vegetables to taste really good. […] Earlier, we didn’t exercise much. But now when there is more time, it is just enjoyable to go and do some training. (Man, informant 8).

Interestingly, despite improved health status, high efficacy beliefs and optimistic attitudes in general, some informants expressed that they could have done more for their health. The informants’ capability of observing themselves with a critical eye stands in contrast to the optimism bias expressed by the individual, described below, representing the *negative case*. A few informants may have made changes initially, but later relapsed, e.g. regarding eating habits. It is also possible that the ability of critical self-evaluation had contributed to monitoring or maintaining health:

I went out for 45 minutes or so, and then I felt that, yeah, now I have done what I should. But maybe I could have done some more. (Woman, informant 6)

The majority of the informants described motivators or stated that they had set goals for themselves. As in the case of decision making, goals were often formulated as avoiding disease or maintaining health. Achievement goals such as losing weight and external goals or motivators were commonly mentioned:

It is this thing, since I have grandchildren, it’s like a carrot so to say. I want to live a long life, I want to live… Life is wonderful and I will do what I can to live a longer life. And feel well. And have the strength. (Woman, informant 5)

### Cognitive and emotional reactions in strong interplay for orchestration of health-related behavioural change

This main theme not only incorporates a wide range of reactions, but also describes how these reactions are highly interlinked in the behavioural change process over time. As described by the categories, the interplay of cognition and emotion is central, from early reactions to risk messages, through the decision-making process to trying to maintain health behaviour. Cognitive and emotional reactions were distinct, but often inseparable.

### Problem-focused coping and encouragement-driven process

Whereas the main theme describes where and when cognitive and emotional reactions interact, the two sub themes describe *how*. Two distinctly different processes were observed. The problem-focused coping process is about acknowledging and fixing the health problem, but the negative starting point does not hinder the individual to be optimistic. A problem-focused attitude, together with strong response efficacy and self-efficacy, can be an effective combination for health improvement, and at the same time imply positive experiences. In particular, this is the case when the informants who have made great effort learn about the improvement.

The other process is driven by encouragement; things were not as bad as expected, getting to know that one is on the right track, or that improvements have been made, encourage the individual to carry on or increase health behaviour. Feedback and encouragement can be enough to maintain or improve health. Although informants had very different emotional reactions to the risk message, the role of self-efficacy and response efficacy were very important in both processes.

### Negative case analysis

In contrast to the other informants included in the analysis, a 64-year old man expressed that he did not believe that the ultrasound results reflected personal risk. Neither did he react emotionally or changed his habits. He had plaque on both sides and red IMT at baseline, but considered the result letter and the nurse call to be *‘just neutral information, nothing I care about’*, and therefore did not follow the nurses’ advice on changing his diet in order to better meet the Nordic nutritional recommendations. The informant expressed that he considered himself to be in good health, and had the attitude that his diet was fine as it was. He stated that he got hypertensive medication due to the result at baseline, something that may explain his improvement. However, he had not understood that high blood pressure is related to atherosclerosis and CVD. Another contrast to the other informants was that this individual made comparisons between his and other’s body shape and health status. This, together with his way of reasoning about the ultrasound result and health risks in general, can be interpreted as a strong optimism bias.

## Discussion

The results showed that cognitive and emotional reactions were highly interlinked in the orchestration of health behaviour, from the initial reactions to the risk communication, across the decision-making process, to maintenance of health behaviour. The main theme, *Cognitive and emotional reactions in strong interplay for orchestration of health-related behavioural change*, might at first glance seem intuitive. However, the strong cognitive focus of early work on determinants of health behaviour might still dominate prevention strategies, even though later findings implicate the need to assess a wide range of factors linked to the indirect effect of emotions on health [17–19]. Also, a meta-review of behaviour change techniques used within cardiovascular prevention to affect self-regulation processes concluded that, among the 15 meta-analyses included, remarkably none of them reported mechanisms of action, the causal processes that underlie successful behavioural change [20]. Thus, descriptions of how cognitive and emotional reactions are highly interlinked in relation to risk communication and prediction of health behaviour do indeed have scientific value.

The two subthemes, *problem-focused coping* and *encouragement-driven process*, were of relevance regarding cognitive and emotional reactions to the pictorial-based intervention, as well as regarding reactions in the process of changing or maintaining behaviour. The problem-focused approach was characterised by the informants’ acknowledgement of their CVD risk, their decisions to do something about their health status, and actions taken to improve their health. This alludes to *active coping* [21] and the similar *problem-focused coping* [22]. Central for these concepts is taking active steps to remove or circumvent a stressor or to amend its effects. Furthermore, this problem-focused approach can be interpreted as a successful behavioural change according to social cognitive models (e.g. the *Health belief model*), since these models focus on perceived consequences and factors such as susceptibility, severity and perceived behavioural control. However, an important distinction is that emotional reactions were central for the informant’s decision-making.

The encouragement-driven process is, to the best of our knowledge, to a less extent described in the literature. A plausible explanation for a positive health message leading to improved health is that it leads to positive affect, which, in turn, influences cognition and behaviour. The informants’ optimistic beliefs regarding possibilities to influence their health show how hope and being future-oriented are highly important, which also alludes to the concept of *empowerment*.

We acknowledge the importance of medication for decreasing CVD risk, and also the importance of adherence to medication, and therefore explored questions also on medication intake and attitudes to medications. However, not much data was generated regarding this, and only a couple of individuals started to taking medication as a result of their baseline visit in VIP or after inclusion in VIPVIZA (one individual also decreased use of medication due to lifestyle modification).

As outlined above, there were several factors of relevance for implementation of health behaviour, including cognitive (e.g. increased awareness of health behaviour) and emotional reactions (e.g. enjoyment of healthy habits) as well as contextual factors (e.g. retirement and social support). This corresponds well to the complex framework proposed by Kiviniemi et al. [19].

Whereas the informants’ emotional reactions varied whether they had a problem-focused or encouragement-driven process, expressions of self-efficacy and response efficacy were very important in both processes. An intuitive interpretation of this is that as long as an emotional reaction was present, the valence of emotion was secondary.

The high extent of efficacy beliefs expressed by the informants independently of perceived severity of the risk message, challenges the assumptions of the *Extended parallel process model* [23] which describes how threat (severity and vulnerability) and efficacy (response efficacy and self-efficacy) interact to produce danger control (self-protective attitudes, intentions, behaviours) or fear control actions (defensive avoidance, denial, reactance). The model assumes that, regardless of perceived efficacy level, there will be no further processing of the message if the threat is low. Our study shows that not only receiving a severe health message, but also receiving a favourable result, can produce behavioural change.

Interestingly, the informants’ descriptions of reactions to the ultrasound result letter seem to be very similar to those of the *HeartAge* study, a qualitative study exploring patients’ perceptions of strategies for communicating CVD risk [24]. As it seems, the two risk communication strategies have in common that they make a strong impression, work as a wake-up call and raise consciousness. Caution should be taken regarding generalisation, but participants in the aforementioned study had an age-span of 27–84 years, which suggests that communicating risk as a graphical presentation of age might be effective also in persons younger and older than the informants in the present study.

From the interviews it became clear that the importance and meaning of the two parts of the intervention differed among informants. For some, the ultrasound result letter was enough, for others the follow-up call was crucial, and yet for others the combination was important. The fact that the emergent design allowed the inclusion criteria to be adapted (to include any or a combination of ultrasound result letter and follow-up call) deepened the analysis of the informants’ understanding of the risk message. Even though the informants’ reactions to the risk message were linked in different ways to the two parts of the intervention, they shared perceptions of the quality and meaning of the message. The message was described as clear, accurate, reliable, engaging and supportive, and the definite nature of the message laid the ground for emotional and cognitive reactions and decision making. In the informants’ descriptions of reactions to the intervention, it was obvious that the potential of the picture is greater than just ‘evoking emotions’, and that the follow-up call can do more than ‘provide support’.

### Methodological considerations

Negotiation of categories among the authors, researcher triangulation within the research group and peer-debriefing with research nurses, as well as the negative case analysis, aimed at increasing the dependability and credibility of the study.

A strength of the study is that the inclusion of men and women with different level of education, living in urban and rural areas and of different health status captured a wide range of experiences. However, since the aim of the study was to reach understanding of characteristics of individuals who managed to reduce their CVD risk when participating in the intervention, a limitation is that reactions from less fortunate groups are not incorporated.

Before the study started, the risk was considered that informants might have difficulties remembering and reporting events three years back in time, which can be a threat to the credibility of the study. However, even though, on a few occasions, informants expressed uncertainty we did not find reason to doubt the accuracy of their memories in general. Rather, the vivid and sometimes very detailed descriptions of reactions may imply not only that the informants remembered their reactions and actions very well, but also that receiving the risk message to a high extent was emotionally engaging, and that the latter might have contributed to the former. Social desirability was considered a minor risk since informants’ stories of lifestyle modifications were coherent with blood samples at the one-year follow-up and ultrasound results at the three-year follow-up.

## Conclusions and implications for research and clinical settings

In conclusion, the present study supports the claim that emotional reactions are important for effective risk communication. Furthermore, the results underscore that an interaction between emotional reactions and efficacy beliefs is important in facilitating behavioural change. The study also highlights the importance of the clear-cut message, and suggests that not only receiving a severe health message, but also receiving a favourable result, can motivate and increase or maintain health behaviour.

Graphical information about atherosclerosis has the potential to conceptualise CVD risk. However, components in studies assessing pictorial risk communication varies considerably. Raw medical images as well as graphical material can be used, and the additional explanations provided can be any combination of verbal communication, numerical risk assessment or written information [3–5,25–27]. Hence, methodological refinement is needed. More research is needed also regarding how to support a successful balance between emotional reactions and efficacy beliefs in personalised risk communication.

Regardless of the modality for communication, understanding *mechanisms of action* is central. Finding ways to promote health behaviour by strengthening efficacy beliefs while taking emotional reactions into consideration should be a future assignment in research and clinical settings.

## Ethical approval

The study was performed in line with the principles of the Declaration of Helsinki and approved by the Umeå Regional Ethics Board (2011-455-31M and 2016-245-32M).

## Consent form

Informed consent regarding participation and publication has been obtained from participants.

## Supplementary Material

Supplemental Material

Supplemental Material
