# Supplementary material for: Cognitive and emotional reactions to pictorial-based risk communication on subclinical atherosclerosis: a qualitative study within the VIPVIZA trial
Source: Scand J Prim Health Care. 2023 Feb 28;41(1):69–80. doi: 10.1080/02813432.2023.2178850 (PMC10088925; doi:10.1080/02813432.2023.2178850)
Supplement: Supplemental Material [file IPRI_A_2178850_SM7554.docx]

**Appendix 2**

**Interview questions**

*The interview was chronologically organized and started with reactions to the intervention at baseline, later in the interview, questions on more recent experiences followed.*

| **Question** | **Focus of question** |
| --- | --- |
| Why did you want to participate in the VIPVIZA study? | Risk perception, motivation |
| How have you experienced receiving pictorial-based health information? | Indication of memory |
| What were your thoughts on cardiovascular diseases, such as high blood pressure, heart attack, or stroke, before taking part in VIPVIZA? | Risk perception, response efficacy |
| During your time in the VIPVIZA study, have you changed your view on the risk of cardiovascular disease in any way | Risk perception, response efficacy, self-efficacy |
| Are you taking any medication for high blood fats, high blood pressure or diabetes?  *(If yes):* For how long have you been taking it, approximately? | Medication |
| So, you received your first ultrasound result letter about three years ago. At that point in time it looked like this…. *(Interviewer here refers to the plaque and IMT status of the informant).* Can you please describe your first reactions when you received the first ultrasound letter? | Emotional and cognitive reactions |
| How was it for you to receive this? Do you remember how you felt right after you had read the letter? Can you describe your thoughts? | Emotional and cognitive reactions |
| Would you describe the health information you received as expected or unexpected? | Emotional and cognitive reactions |
| If you look back at the time after the first ultrasound message, do you think it affected you emotionally in any way? | Emotional reactions |
| Did you talk to anyone around you about your health? How did they react? | Social support, indication of  memory |
| After you received the first letter, you also received a phone call from a nurse.  How did you feel about discussing the ultrasound result with the nurse?  Can you describe your feelings and thoughts after the conversation? | Indication of memory, emotional and cognitive reactions, response efficacy |
| Receiving information about one´s health can be perceived as frightening or threatening, but one can also perceive new opportunities to influence one´s health in a positive direction.  Can you please describe how it was to receive the ultrasound message and talk to the nurse, in terms of threats and/or opportunities regarding your health? | Perceived severity, optimism /pessimism, response efficacy |
| Did the first ultrasound result letter and nurse call lead to you changing anything, big or small, in your eating habits/exercise habits/tobacco/alcohol consumption (asked respectively for each habit)? | Action |
| *(If the informant answered yes to any of the questions regarding changed health behaviors):* How come you did… /changed… Why is it that you came to focus on that particular aspect, do you think? | Action, self-efficacy, response efficacy, motivation, social support |
| (Question especially to those who describe that they have made extensive lifestyle changes): Many want to improve their health, but at the same time it can be difficult to change habits. Why do you think you succeeded in making lifestyle changes? Why do you think you managed to maintain these changes, that you have managed to keep up with the changed eating habits/exercise habits etc? Have you changed your habits earlier in life? | Action, response efficacy, emotional and cognitive reactions |
| (If the informant is taking medication): You told me earlier that you are taking medication. What are your thoughts on the doctor prescribing medicine to you?  What are your thoughts on taking medication as a daily routine? | Action, response efficacy, emotional and cognitive reaction |
| Do you remember how you looked upon your chances of affecting your risk of cardiovascular disease through lifestyle changes such as changes in diet and exercise habits after the first ultrasound examination? | Response efficacy |
| From a practical point of view, what possibilities and what time frames did you have to make lifestyle changes? | Barriers |
| Do you recall how you looked upon your ability to make lifestyle changes? | Self-efficacy |
| Can you describe how you may have set goals in relation to your health, for example regarding weight, smoking or exercise habits? (If the informant describes setting a goal): How come you…? What would motivate you to set goals / set other goals to improve your health? | Motivation, action, response efficacy |
| Your test result one year after the first ultrasound showed that your health had improved. Why so, do you think?  (To informants who described lifestyle changes): Your latest ultrasound result was… which is interpreted positively. When you… changed your diet / stopped smoking / etc. did you then believe that your efforts would pay off? | Response efficacy |
| As I said, your test results after the first year had improved. And now the ultrasound… *(repeating the informant’s new plaque and IMT status).* How do you see your opportunities to influence your health for further improvement through lifestyle changes such as diet and exercise? | Response efficacy |
| You have recently received your second ultrasound result. What was it like to receive it? Do you remember how you felt right after you had read the letter? | Emotional reactions |
| Can you describe your thoughts about the ultrasound result right now? Would you say that this result was expected or unexpected to you? | Emotional and cognitive reactions |
| How do you look upon your opportunities to continue to positively affect your risk of cardiovascular disease through lifestyle changes such as changes in eating and exercise habits? | Response efficacy |
| Can you please describe how you look upon your opportunities to live a healthy life today? | Barriers, self-efficacy |
| Is there anything that would make it easier for you to live an even healthier life in the future? | Barriers |
| Is there anything that hinders or reduces your motivation for lifestyle change? | Motivation, barriers, self-efficacy |
| Is there anything that has been of particular importance for your motivation to start making lifestyle changes during your time in Vipviza? | Motivation |
| Is there anything that has been of particular importance for your motivation to maintain lifestyle changes/continue with your… jogging etc. during your time in the Vipviza study? | Perceived benefit, motivation |
| Do you think that you have benefitted in any way from participating in the VIPVIZA study? | Perceived benefit |
