# Supplementary material for: Cognitive and emotional reactions to pictorial-based risk communication on subclinical atherosclerosis: a qualitative study within the VIPVIZA trial
Source: Scand J Prim Health Care. 2023 Feb 28;41(1):69–80. doi: 10.1080/02813432.2023.2178850 (PMC10088925; doi:10.1080/02813432.2023.2178850)
Supplement: Supplemental Material [file IPRI_A_2178850_SM5773.docx]

# Appendix 1

Written information is provided in the result letter, apart from graphical information. Since graphical elements of the result letter is presented as a picture in the article (Figure 1), only the text is presented below. The general information under the heading “What can an ultrasound examination show?” is given to all participants. The graphical information on plaque and IMT is personalized and based on the result of the ultrasound assessment. Explanations regarding the colour code for IMT, and the advice for staying healthy, are adapted to the participants’ ultrasound results. That is, a person with a favourable atherosclerosis status (green-coded result message) does not read the advice aimed at a person with pronounced atherosclerosis (red-coded result message). However, in this appendix, we present the advice for each colour-coded risk message. In Swedish, the word “åderförkalkning” is used throughout the result letter for atherosclerosis. Compared to atherosclerosis, this word is less of a medical term, rather, it is used in every-day language among people, and means approximately “ageing, clogging vessels by chalk accumulation”

**What can an ultrasound examination show?**

The ultrasound examination measures two layers of the carotid artery wall (intima and media), the so-called IMT value.

A thickening of these layers is the first sign of atherosclerosis. Accumulation of calcium in the vessel wall, known as plaques, can also be detected and is a sign of more pronounced atherosclerosis, even if the wall thickness is not increased.

Atherosclerosis develops slowly from a young age, and is linked to lifestyle, heredity, blood pressure, blood lipids, blood sugar and weight. This was measured and discussed with you at your health check-up.

Atherosclerosis increases the risk of heart attack, stroke and shortened life expectancy due to cardiovascular disease. On the positive side, the development of atherosclerosis is a dynamic process that **you can influence** and thus also your risk of disease.

**Atherosclerosis can be slowed down and even reversed.**

This is accomplished through a healthy lifestyle including being physically active and eating healthy, and avoiding smoking and stress. If you are prescribed medication for high blood pressure, high blood lipids or diabetes, it is important to take your medication in order for the treatment to be effective.

**Blood vessels in the neck**

**
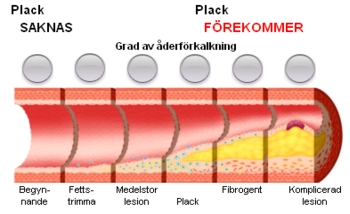
**

**Normal Developing Plaque Pronounced
blood vessel atherosclerosis atherosclerosis**

**NOTE! This is not your blood vessel, but a schematic picture of how atherosclerosis can develop over time.**

People with severe constriction of the blood vessels who need urgent treatment are referred for further investigation, and do not receive this message. You do not belong to this group.

(Graphical information on plaque and IMT is provided here)

**On the last page, based on the results of your ultrasound examination, there are recommendations on what you can do to promote your own health.**

In people with any risk factor for cardiovascular disease (such as smoking, diabetes, high blood lipids or family history of cardiovascular disease), plaque is:

- uncommon at the age of 40,

- present in 10-15% at age 50 and

- present in 30-40%, i.e. quite common, at the age of 60.

**What can you do yourself to stay healthy?**

The ultrasound examination of your carotid arteries shows the thickness of the vessel wall and whether there are plaques. What to do is determined by the most pronounced change found in your vessels. If you have a thickened vessel wall and/or plaque on one side, this will determine what to do, even if there are less pronounced changes on the other side.

**THE ULTRASOUND EXAMINATION OF YOUR CAROTID ARTERIES CAN BE SUMMARISED AS FOLLOWS:**

The color chart takes your age into account.

| **Green** | |  | No or less pronounced signs of atherosclerosis.  It is important **TO MAINTAIN A HEALTHY LIFE STYLE.** |
| --- | --- | --- | --- |
|  | |  |  |
| **Yellow** | **Orange** |  | Moderate signs of atherosclerosis.  Yellow is under and orange is over the average for your age.  It is important **TO SLOW DOWN OR REVERSE THE DEVELOPMENT OF ATHEROSCLEROSIS THROUGH A HEALTHY LIFESTYLE**. |
|  | |  |  |
| **Red** | |  | Pronounced signs of atherosclerosis.  It is important **TO SLOW DOWN OR REVERSE THE DEVELOPMENT OF ATHEROSCLEROSIS THROUGH HEALTHY LIVESTYLE.** |

**REGARDLESS OF OBTAINED COLOUR SIGNAL, FOR INDIVIDUALS TAKING MEDICATION FOR:**

**- HIGH BLOOD LIPIDS
- HIGH BLOOD PRESSURE** or **- DIABETES**

It is important also to **TAKE YOUR MEDICATION**, in order for the medical treatment to be effective.

**Useful information to be found on the internet**

The following links will provide you with a lot of good information and also additional links about healthy lifestyles and tips and support for changing your habits.

[**www.sundkurs.se**](http://www.sundkurs.se) [**www.halsa2020.se**](http://www.halsa2020.se)
